# Aqua­(picolinato *N*-oxide-κ^2^ *O* ^1^,*O* ^2^)(pyridine-2,6-dicarboxyl­ato-κ^3^ *O*,*N*,*O*′)iron(III) monohydrate

**DOI:** 10.1107/S1600536808030663

**Published:** 2008-09-27

**Authors:** Dongdong Han, Dong’e Wang

**Affiliations:** a Management Department of Zhejiang Pharmaceutical College, Ningbo, Zhejiang 315100, People’s Republic of China; b Department of Chemistry, Kashgar Teachers College, Kashgar, Xinjiang 844000, People’s Republic of China

## Abstract

In the title compound, [Fe(C_6_H_4_NO_3_)(C_7_H_3_NO_4_)(H_2_O)]·H_2_O, the Fe^III^ ion is coordinated by two O and one N atoms from a pyridine-2,6-dicarboxyl­ate ligand, by two O atoms from a picolinate *N*-oxide ligand and by one water O atom in a distorted octa­hedral geometry [Fe—O = 1.940 (3)–2.033 (3) Å and Fe—N = 2.057 (4) Å]. In the crystal structure, the coordinated and solvent water mol­ecules contribute to the formation of O—H⋯O hydrogen bonds, which link the mol­ecules into layers parallel to the *ab* plane.

## Related literature

For related crystal structures, see: Lainé *et al.* (1995 ▶); Wu *et al.* (2007 ▶).
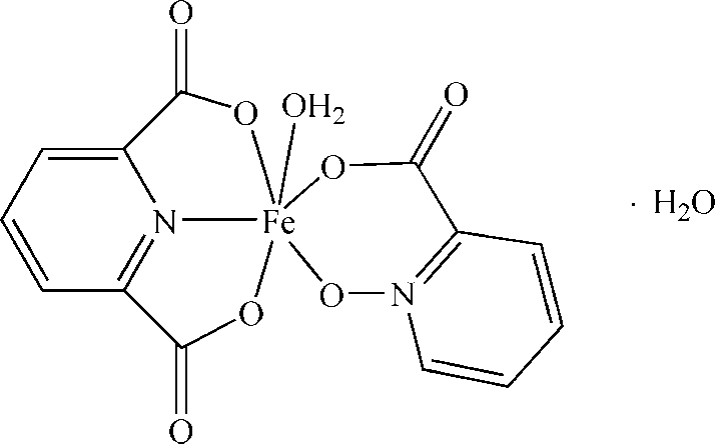


## Experimental

### 

#### Crystal data

[Fe(C_6_H_4_NO_3_)(C_7_H_3_NO_4_)(H_2_O)]·H_2_O
*M* *_r_* = 395.09
Triclinic, 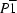

*a* = 6.6023 (13) Å
*b* = 7.7256 (16) Å
*c* = 15.520 (3) Å
α = 102.585 (4)°
β = 95.801 (4)°
γ = 105.743 (4)°
*V* = 732.7 (3) Å^3^
*Z* = 2
Mo *K*α radiation
μ = 1.09 mm^−1^
*T* = 293 (2) K
0.16 × 0.14 × 0.12 mm

#### Data collection

Bruker SMART CCD area-detector diffractometer
Absorption correction: multi-scan (*SADABS*; Sheldrick, 2000 ▶) *T*_min_ = 0.84, *T*_max_ = 0.87
3915 measured reflections
2749 independent reflections
1667 reflections with *I* > 2σ(*I*)
*R*_int_ = 0.047

#### Refinement

*R*[*F* ^2^ > 2σ(*F* ^2^)] = 0.060
*wR*(*F* ^2^) = 0.096
*S* = 0.85
2749 reflections
238 parameters
4 restraints
H atoms treated by a mixture of independent and constrained refinement
Δρ_max_ = 0.49 e Å^−3^
Δρ_min_ = −0.36 e Å^−3^

### 

Data collection: *SMART* (Bruker, 2001 ▶); cell refinement: *SAINT-Plus* (Bruker, 2001 ▶); data reduction: *SAINT-Plus*; program(s) used to solve structure: *SHELXS97* (Sheldrick, 2008 ▶); program(s) used to refine structure: *SHELXL97* (Sheldrick, 2008 ▶); molecular graphics: *SHELXTL* (Sheldrick, 2008 ▶); software used to prepare material for publication: *SHELXTL*.

## Supplementary Material

Crystal structure: contains datablocks I, global. DOI: 10.1107/S1600536808030663/cv2445sup1.cif

Structure factors: contains datablocks I. DOI: 10.1107/S1600536808030663/cv2445Isup2.hkl

Additional supplementary materials:  crystallographic information; 3D view; checkCIF report

## Figures and Tables

**Table 1:** Hydrogen-bond geometry (Å, °)

*D*—H⋯*A*	*D*—H	H⋯*A*	*D*⋯*A*	*D*—H⋯*A*
O8—H8*A*⋯O2^i^	0.85 (4)	1.81 (4)	2.637 (5)	164 (4)
O8—H8*B*⋯O9^ii^	0.83 (3)	1.79 (4)	2.571 (5)	157 (5)
O9—H9*A*⋯O3^iii^	0.87 (5)	1.88 (5)	2.730 (5)	167 (5)
O9—H9*B*⋯O5	0.86 (5)	1.97 (6)	2.821 (5)	173 (4)

Symmetry codes: (i) 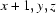; (ii) 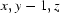; (iii) 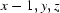.

**Fig. 1.**
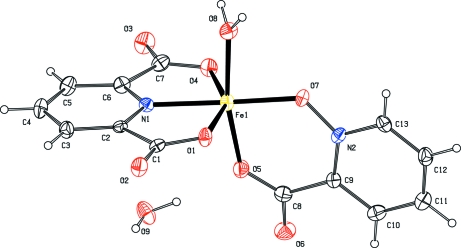
The molecular structure of (I) showing the atomic numbering and 50% probability displacement ellipsoids.

**Fig. 2.**
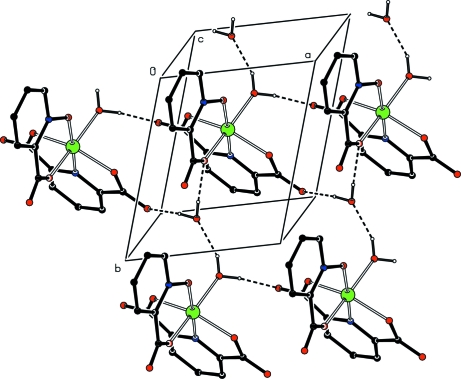
A portion of the crystal packing showing H-bonds as dashed lines.

## supplementary crystallographic information

### Comment

Recently, the  2D zinc(II) and 1D copper(II) complexes with pyridine-2,6-dicarboxylic acid N-oxide and dicarboxylato ligands were reported by Wu *et al.* (2007). As a contribution to this area, we report the crystal structure of the title compound, (I).

In (I) (Fig. 1), the  Fe^III^ ion is coordinated by two O and one N atoms from pyridine-2,6-dicarboxylato ligand, two O atoms from picolinato-N-oxide ligand, and one water molecule in a distorted octahedral geometry. Atoms O1, O4, O7 and N1 lie in equatorial plane, with the O1—N1—O4—O7 torsion angle of 1.94 (15)°, while Fe1 deviates from the equatorial plane at 0.057 Å. Atoms O5 and O8 occupy the axial sites with the angle O5—Fe1—O8 of 167.93 (14)°. The bond lengths and angles in (I) are similar to those in the related Fe^III^ complex (Lainé *et al.*,1995).

In the crystal, the coordinated and crystalline water molecules contribute to the formation of O—H···O hydrogen bonds (Table 1, Fig. 2), which link the molecules into the layers parallel to *ab* plane.

### Experimental

A mixture of Fe_2_(SO_4_)_3_(0.5 mmol), pyco (0.5 mmol), pydc (0.50 mmol), and H_2_O (3.00 ml), was placed in a Parr Teflon-lined stainless steel vessel (10 ml), and then the vessel was sealed and heated at 393 K for 3 d. After the mixture was slowly cooled to room temperature, several red crystals of (I) were obtained.

### Refinement

C-bound H atoms were introduced at calculated positions (C—H 0.93 Å) and refined using a riding model, with *U*_iso_(H) = 1.2*U*_eq_(C). H atoms of water molecules were located in a difference Fourier map and refined with O—H and H···H distance restraints of 0.85 (3) and 1.39 (3) Å, respectively, and *U*_iso_(H) = 1.5*U*_eq_(O).

### Crystal data

**Table:**

[Fe(C_6_H_4_NO_3_)(C_7_H_3_NO_4_)(H_2_O)]·H_2_O	*Z* = 2
*M**_r_* = 395.09	*F*(000) = 402
Triclinic, *P*1	*D*_x_ = 1.791 Mg m^−^^3^
Hall symbol: -P 1	Mo *K*α radiation, λ = 0.71073 Å
*a* = 6.6023 (13) Å	Cell parameters from 544 reflections
*b* = 7.7256 (16) Å	θ = 2.7–20.0°
*c* = 15.520 (3) Å	µ = 1.09 mm^−^^1^
α = 102.585 (4)°	*T* = 293 K
β = 95.801 (4)°	Block, red
γ = 105.743 (4)°	0.16 × 0.14 × 0.12 mm
*V* = 732.7 (3) Å^3^	

### Data collection

**Table:**

Bruker SMART CCD area-detector diffractometer	2749 independent reflections
Radiation source: fine-focus sealed tube	1667 reflections with *I* > 2σ(*I*)
graphite	*R*_int_ = 0.047
φ and ω scans	θ_max_ = 25.8°, θ_min_ = 2.7°
Absorption correction: multi-scan (SADABS; Sheldrick, 2000)	*h* = −8→7
*T*_min_ = 0.84, *T*_max_ = 0.87	*k* = −9→9
3915 measured reflections	*l* = −18→17

### Refinement

**Table:**

Refinement on *F*^2^	Primary atom site location: structure-invariant direct methods
Least-squares matrix: full	Secondary atom site location: difference Fourier map
*R*[*F*^2^ > 2σ(*F*^2^)] = 0.060	Hydrogen site location: inferred from neighbouring sites
*wR*(*F*^2^) = 0.096	H atoms treated by a mixture of independent and constrained refinement
*S* = 0.85	*w* = 1/[σ^2^(*F*_o_^2^) + (0.0145*P*)^2^] where *P* = (*F*_o_^2^ + 2*F*_c_^2^)/3
2749 reflections	(Δ/σ)_max_ = 0.001
238 parameters	Δρ_max_ = 0.49 e Å^−^^3^
4 restraints	Δρ_min_ = −0.36 e Å^−^^3^

### Special details

**Table:**

Geometry. All e.s.d.'s (except the e.s.d. in the dihedral angle between two l.s. planes) are estimated using the full covariance matrix. The cell e.s.d.'s are taken into account individually in the estimation of e.s.d.'s in distances, angles and torsion angles; correlations between e.s.d.'s in cell parameters are only used when they are defined by crystal symmetry. An approximate (isotropic) treatment of cell e.s.d.'s is used for estimating e.s.d.'s involving l.s. planes.
Refinement. Refinement of *F*^2^ against ALL reflections. The weighted *R*-factor *wR* and goodness of fit *S* are based on *F*^2^, conventional *R*-factors *R* are based on *F*, with *F* set to zero for negative *F*^2^. The threshold expression of *F*^2^ > σ(*F*^2^) is used only for calculating *R*-factors(gt) *etc*. and is not relevant to the choice of reflections for refinement. *R*-factors based on *F*^2^ are statistically about twice as large as those based on *F*, and *R*- factors based on ALL data will be even larger.

### Fractional atomic coordinates and isotropic or equivalent isotropic displacement parameters (Å^2^)

**Table:**

	*x*	*y*	*z*	*U*_iso_*/*U*_eq_	
Fe1	0.47020 (11)	0.38418 (9)	0.22899 (5)	0.0363 (2)	
N1	0.4982 (6)	0.5619 (5)	0.3522 (2)	0.0317 (10)	
N2	0.3098 (6)	0.1709 (5)	0.0392 (3)	0.0358 (10)	
O1	0.1839 (4)	0.2941 (4)	0.2682 (2)	0.0404 (9)	
O4	0.7711 (5)	0.5595 (4)	0.2520 (2)	0.0439 (9)	
O5	0.3665 (5)	0.5275 (4)	0.1559 (2)	0.0497 (10)	
O8	0.5897 (5)	0.2107 (4)	0.2796 (2)	0.0450 (10)	
H8A	0.722 (5)	0.241 (7)	0.300 (3)	0.067*	
H8B	0.553 (8)	0.098 (4)	0.276 (4)	0.067*	
C1	0.1473 (7)	0.3772 (6)	0.3417 (3)	0.0344 (12)	
C2	0.3323 (7)	0.5401 (6)	0.3952 (3)	0.0293 (11)	
C3	0.3435 (7)	0.6559 (6)	0.4769 (3)	0.0408 (13)	
H3	0.2269	0.6422	0.5066	0.049*	
C4	0.5325 (8)	0.7933 (6)	0.5138 (3)	0.0467 (14)	
H4	0.5454	0.8734	0.5696	0.056*	
C5	0.7012 (7)	0.8134 (6)	0.4693 (3)	0.0448 (14)	
H5	0.8294	0.9062	0.4945	0.054*	
C6	0.6799 (7)	0.6952 (6)	0.3869 (3)	0.0390 (13)	
C7	0.8408 (8)	0.6911 (7)	0.3244 (4)	0.0444 (14)	
C8	0.3104 (8)	0.5009 (7)	0.0716 (4)	0.0415 (13)	
C9	0.2594 (7)	0.3099 (6)	0.0109 (3)	0.0322 (12)	
C10	0.1569 (7)	0.2692 (7)	−0.0765 (3)	0.0444 (14)	
H10	0.1215	0.3625	−0.0979	0.053*	
C11	0.1064 (8)	0.0935 (7)	−0.1323 (4)	0.0501 (15)	
H11	0.0391	0.0679	−0.1913	0.060*	
C12	0.1560 (8)	−0.0406 (7)	−0.1002 (4)	0.0462 (14)	
H12	0.1214	−0.1602	−0.1373	0.055*	
C13	0.2553 (7)	−0.0048 (6)	−0.0149 (3)	0.0377 (13)	
H13	0.2863	−0.0996	0.0068	0.045*	
O2	−0.0192 (5)	0.3380 (4)	0.3721 (2)	0.0479 (10)	
O3	1.0189 (5)	0.8053 (5)	0.3461 (2)	0.0594 (11)	
O6	0.2880 (6)	0.6231 (5)	0.0361 (3)	0.0600 (11)	
O7	0.4227 (5)	0.1917 (4)	0.1189 (2)	0.0463 (9)	
O9	0.3706 (6)	0.8722 (5)	0.2653 (3)	0.0608 (12)	
H9A	0.249 (6)	0.839 (7)	0.283 (4)	0.091*	
H9B	0.366 (9)	0.771 (5)	0.228 (3)	0.091*	

### Atomic displacement parameters (Å^2^)

**Table:**

	*U*^11^	*U*^22^	*U*^33^	*U*^12^	*U*^13^	*U*^23^
Fe1	0.0375 (4)	0.0354 (4)	0.0324 (4)	0.0069 (3)	0.0085 (3)	0.0053 (3)
N1	0.027 (2)	0.031 (2)	0.036 (3)	0.0067 (18)	0.0058 (19)	0.0084 (19)
N2	0.031 (2)	0.046 (3)	0.028 (3)	0.007 (2)	0.0038 (19)	0.011 (2)
O1	0.0294 (19)	0.040 (2)	0.035 (2)	−0.0034 (15)	0.0042 (16)	−0.0056 (16)
O4	0.033 (2)	0.049 (2)	0.046 (2)	0.0054 (16)	0.0136 (17)	0.0112 (18)
O5	0.069 (3)	0.042 (2)	0.039 (2)	0.0211 (18)	0.004 (2)	0.0075 (18)
O8	0.042 (2)	0.035 (2)	0.051 (3)	0.0033 (19)	−0.0009 (19)	0.0104 (19)
C1	0.029 (3)	0.040 (3)	0.033 (3)	0.009 (2)	−0.001 (2)	0.012 (2)
C2	0.022 (3)	0.032 (3)	0.032 (3)	0.008 (2)	0.000 (2)	0.008 (2)
C3	0.037 (3)	0.047 (3)	0.035 (3)	0.010 (2)	0.009 (2)	0.004 (3)
C4	0.057 (4)	0.041 (3)	0.032 (3)	0.006 (3)	0.003 (3)	0.000 (3)
C5	0.038 (3)	0.037 (3)	0.043 (4)	−0.008 (2)	−0.001 (3)	0.005 (3)
C6	0.036 (3)	0.031 (3)	0.045 (4)	0.002 (2)	0.005 (3)	0.010 (3)
C7	0.040 (3)	0.049 (3)	0.043 (4)	0.007 (3)	0.004 (3)	0.019 (3)
C8	0.033 (3)	0.044 (3)	0.049 (4)	0.010 (3)	0.011 (3)	0.015 (3)
C9	0.031 (3)	0.032 (3)	0.033 (3)	0.005 (2)	0.011 (2)	0.010 (2)
C10	0.035 (3)	0.054 (4)	0.048 (4)	0.011 (3)	0.008 (3)	0.023 (3)
C11	0.050 (4)	0.053 (4)	0.040 (4)	0.010 (3)	0.002 (3)	0.009 (3)
C12	0.036 (3)	0.049 (3)	0.042 (4)	0.007 (3)	0.006 (3)	−0.004 (3)
C13	0.032 (3)	0.033 (3)	0.045 (4)	0.007 (2)	0.013 (3)	0.005 (3)
O2	0.027 (2)	0.061 (2)	0.044 (2)	0.0007 (16)	0.0073 (17)	0.0029 (18)
O3	0.034 (2)	0.063 (2)	0.065 (3)	−0.0095 (18)	0.0098 (19)	0.013 (2)
O6	0.071 (3)	0.051 (2)	0.065 (3)	0.021 (2)	0.007 (2)	0.028 (2)
O7	0.054 (2)	0.055 (2)	0.026 (2)	0.0208 (18)	−0.0004 (18)	0.0015 (17)
O9	0.060 (3)	0.034 (2)	0.079 (3)	0.005 (2)	0.027 (2)	0.000 (2)

### Geometric parameters (Å, °)

**Table:**

Fe1—O7	1.939 (3)	C3—H3	0.9300
Fe1—O5	1.945 (4)	C4—C5	1.360 (6)
Fe1—O8	1.986 (4)	C4—H4	0.9300
Fe1—O4	2.023 (3)	C5—C6	1.369 (6)
Fe1—O1	2.032 (3)	C5—H5	0.9300
Fe1—N1	2.055 (4)	C6—C7	1.511 (6)
N1—C6	1.321 (5)	C7—O3	1.226 (5)
N1—C2	1.331 (5)	C8—O6	1.226 (6)
N2—O7	1.332 (5)	C8—C9	1.496 (6)
N2—C9	1.352 (5)	C9—C10	1.382 (6)
N2—C13	1.362 (5)	C10—C11	1.374 (6)
O1—C1	1.262 (5)	C10—H10	0.9300
O4—C7	1.285 (5)	C11—C12	1.344 (7)
O5—C8	1.279 (6)	C11—H11	0.9300
O8—H8A	0.85 (3)	C12—C13	1.351 (7)
O8—H8B	0.83 (3)	C12—H12	0.9300
C1—O2	1.228 (5)	C13—H13	0.9300
C1—C2	1.507 (6)	O9—H9A	0.87 (3)
C2—C3	1.365 (6)	O9—H9B	0.86 (3)
C3—C4	1.372 (6)		
			
O7—Fe1—O5	86.72 (14)	C2—C3—H3	121.1
O7—Fe1—O8	82.18 (14)	C4—C3—H3	121.1
O5—Fe1—O8	167.93 (15)	C5—C4—C3	120.6 (4)
O7—Fe1—O4	110.22 (14)	C5—C4—H4	119.7
O5—Fe1—O4	91.59 (14)	C3—C4—H4	119.7
O8—Fe1—O4	87.85 (14)	C4—C5—C6	119.1 (4)
O7—Fe1—O1	98.80 (13)	C4—C5—H5	120.4
O5—Fe1—O1	93.23 (14)	C6—C5—H5	120.4
O8—Fe1—O1	93.13 (14)	N1—C6—C5	120.0 (4)
O4—Fe1—O1	150.80 (13)	N1—C6—C7	111.0 (4)
O7—Fe1—N1	172.69 (15)	C5—C6—C7	129.1 (4)
O5—Fe1—N1	97.84 (14)	O3—C7—O4	126.8 (5)
O8—Fe1—N1	93.69 (15)	O3—C7—C6	119.7 (5)
O4—Fe1—N1	75.50 (13)	O4—C7—C6	113.5 (4)
O1—Fe1—N1	75.31 (13)	O6—C8—O5	123.9 (5)
C6—N1—C2	121.5 (4)	O6—C8—C9	116.3 (5)
C6—N1—Fe1	119.4 (3)	O5—C8—C9	119.8 (5)
C2—N1—Fe1	119.1 (3)	N2—C9—C10	117.5 (4)
O7—N2—C9	124.5 (4)	N2—C9—C8	121.7 (5)
O7—N2—C13	113.8 (4)	C10—C9—C8	120.8 (5)
C9—N2—C13	121.6 (4)	C11—C10—C9	121.3 (5)
C1—O1—Fe1	120.7 (3)	C11—C10—H10	119.4
C7—O4—Fe1	120.6 (3)	C9—C10—H10	119.4
C8—O5—Fe1	133.6 (3)	C12—C11—C10	118.8 (5)
Fe1—O8—H8A	121 (3)	C12—C11—H11	120.6
Fe1—O8—H8B	136 (4)	C10—C11—H11	120.6
H8A—O8—H8B	101 (5)	C11—C12—C13	121.2 (5)
O2—C1—O1	126.8 (4)	C11—C12—H12	119.4
O2—C1—C2	118.9 (4)	C13—C12—H12	119.4
O1—C1—C2	114.3 (4)	C12—C13—N2	119.5 (5)
N1—C2—C3	121.0 (4)	C12—C13—H13	120.2
N1—C2—C1	110.7 (4)	N2—C13—H13	120.2
C3—C2—C1	128.3 (4)	N2—O7—Fe1	129.5 (3)
C2—C3—C4	117.8 (4)	H9A—O9—H9B	101 (5)
			
O7—Fe1—N1—C6	142.0 (11)	C3—C4—C5—C6	0.3 (8)
O5—Fe1—N1—C6	−89.7 (4)	C2—N1—C6—C5	0.7 (8)
O8—Fe1—N1—C6	86.7 (4)	Fe1—N1—C6—C5	−179.4 (4)
O4—Fe1—N1—C6	−0.1 (4)	C2—N1—C6—C7	−179.3 (4)
O1—Fe1—N1—C6	179.0 (4)	Fe1—N1—C6—C7	0.6 (5)
O7—Fe1—N1—C2	−38.1 (14)	C4—C5—C6—N1	−1.0 (8)
O5—Fe1—N1—C2	90.2 (4)	C4—C5—C6—C7	179.0 (5)
O8—Fe1—N1—C2	−93.4 (4)	Fe1—O4—C7—O3	179.8 (4)
O4—Fe1—N1—C2	179.8 (4)	Fe1—O4—C7—C6	1.0 (6)
O1—Fe1—N1—C2	−1.1 (3)	N1—C6—C7—O3	−179.9 (5)
O7—Fe1—O1—C1	176.8 (4)	C5—C6—C7—O3	0.1 (9)
O5—Fe1—O1—C1	−96.0 (4)	N1—C6—C7—O4	−1.0 (6)
O8—Fe1—O1—C1	94.3 (4)	C5—C6—C7—O4	179.0 (5)
O4—Fe1—O1—C1	3.1 (5)	Fe1—O5—C8—O6	−165.3 (3)
N1—Fe1—O1—C1	1.3 (4)	Fe1—O5—C8—C9	17.2 (7)
O7—Fe1—O4—C7	−175.7 (4)	O7—N2—C9—C10	174.6 (4)
O5—Fe1—O4—C7	97.2 (4)	C13—N2—C9—C10	−2.3 (6)
O8—Fe1—O4—C7	−94.9 (4)	O7—N2—C9—C8	−5.9 (7)
O1—Fe1—O4—C7	−2.3 (5)	C13—N2—C9—C8	177.2 (4)
N1—Fe1—O4—C7	−0.5 (4)	O6—C8—C9—N2	169.3 (4)
O7—Fe1—O5—C8	−4.4 (5)	O5—C8—C9—N2	−13.0 (7)
O8—Fe1—O5—C8	18.6 (10)	O6—C8—C9—C10	−11.3 (7)
O4—Fe1—O5—C8	105.7 (5)	O5—C8—C9—C10	166.5 (5)
O1—Fe1—O5—C8	−103.1 (5)	N2—C9—C10—C11	0.6 (7)
N1—Fe1—O5—C8	−178.7 (5)	C8—C9—C10—C11	−178.9 (4)
Fe1—O1—C1—O2	179.0 (4)	C9—C10—C11—C12	0.8 (8)
Fe1—O1—C1—C2	−1.2 (5)	C10—C11—C12—C13	−0.5 (8)
C6—N1—C2—C3	0.4 (7)	C11—C12—C13—N2	−1.2 (8)
Fe1—N1—C2—C3	−179.5 (3)	O7—N2—C13—C12	−174.5 (4)
C6—N1—C2—C1	−179.2 (4)	C9—N2—C13—C12	2.7 (7)
Fe1—N1—C2—C1	0.8 (5)	C9—N2—O7—Fe1	22.0 (6)
O2—C1—C2—N1	−180.0 (4)	C13—N2—O7—Fe1	−160.9 (3)
O1—C1—C2—N1	0.2 (6)	O5—Fe1—O7—N2	−15.4 (4)
O2—C1—C2—C3	0.4 (8)	O8—Fe1—O7—N2	169.3 (4)
O1—C1—C2—C3	−179.4 (5)	O4—Fe1—O7—N2	−105.9 (4)
N1—C2—C3—C4	−1.1 (7)	O1—Fe1—O7—N2	77.3 (4)
C1—C2—C3—C4	178.5 (5)	N1—Fe1—O7—N2	113.4 (12)
C2—C3—C4—C5	0.7 (8)		

### Hydrogen-bond geometry (Å, °)

**Table:**

*D*—H···*A*	*D*—H	H···*A*	*D*···*A*	*D*—H···*A*
O8—H8A···O2^i^	0.85 (4)	1.81 (4)	2.637 (5)	164 (4)
O8—H8B···O9^ii^	0.83 (3)	1.79 (4)	2.571 (5)	157 (5)
O9—H9A···O3^iii^	0.87 (5)	1.88 (5)	2.730 (5)	167 (5)
O9—H9B···O5	0.86 (5)	1.97 (6)	2.821 (5)	173 (4)

Symmetry codes: (i) *x*+1, *y*, *z*; (ii) *x*, *y*−1, *z*; (iii) *x*−1, *y*, *z*.
